# Modified Stancu operators based on inverse Polya Eggenberger distribution

**DOI:** 10.1186/s13660-017-1328-9

**Published:** 2017-03-06

**Authors:** Sheetal Deshwal, PN Agrawal, Serkan Araci

**Affiliations:** 1 Indian Institute of Techology Roorkee, Roorkee, 247667 India; 2 Department of Economics, Faculty of Economics, Administrative and Social Sciences, Hasan Kalyoncu University, Gaziantep, 27410 Turkey

**Keywords:** 41A36, 41A25, Baskakov operator, Ditzian-Totik modulus of smoothness, rate of convergence, Voronovskaja-type theorem

## Abstract

In this paper, we construct a sequence of modified Stancu-Baskakov operators for a real valued function bounded on $[0,\infty)$, based on a function $\tau(x)$. This function $\tau(x)$ is infinite times continuously differentiable on $[0,\infty)$ and satisfy the conditions $\tau (0)=0,~\tau ^{\prime}(x)>0$ and $\tau^{\prime\prime}(x)$ is bounded for all $x\in {}[0,\infty)$. We study the degree of approximation of these operators by means of the Peetre K-functional and the Ditzian-Totik modulus of smoothness. The quantitative Voronovskaja-type theorems are also established in terms of the first order Ditzian-Totik modulus of smoothness.

## Introduction

In 1923, Eggenberger and Pólya [1] were the first to introduce Pólya-Eggenberger distribution. After that, in 1969, Johnson and Kotz [2] gave a short discussion of Pólya-Eggenberger distribution.

The Pólya-Eggenberger distribution *X* [2] is defined by

1.1 $$\begin{aligned} &Pr(X=k)={\binom{n}{k}}\frac{a(a+s)\cdots(a+(u-1)s)b(b+s)\cdots (b+(n-u-1)s)}{(a+b)(a+b+s)\cdots(a+b+(n-1)s)}, \\ &\quad k=0,1,2,\ldots, n. \end{aligned}$$

The inverse Pólya-Eggenberger distribution *N* is defined by

1.2 $$\begin{aligned} &Pr(N=n+k)={\binom{(n+k-1)}{k}}\frac{a(a+s)\cdots(a+(n-1)s)b(b+s)\cdots (b+(k-1)s)}{(a+b)(a+b+s)\cdots(a+b+(n+k-1)s)}, \\ &\quad k=0,1,2,\ldots, n. \end{aligned}$$

In 1970, Stancu [3] introduced a generalization of the Baskakov operators based on inverse Pólya-Eggenberger distribution for a real valued bounded function on $[0,\infty)$, defined by

1.3 $$\begin{aligned} V_{n}^{[\alpha]}(f;x) =& \sum _{k=0}^{\infty} v_{n,k}(x,\alpha)f \biggl( \frac{k}{n} \biggr) =\sum_{k=0}^{\infty} {\binom{n+k-1}{k}} \frac{1^{[n,-\alpha]}x^{[k ,-\alpha]}}{(1+x)^{[n+k,-\alpha]}}f \biggl(\frac{k}{n} \biggr), \end{aligned}$$

where *α* is a non-negative parameter which may depend only on $n\in \mathbb{N}$ and $a^{[n,h]}=a(a-h)(a-2h)\cdots(a-(n-1)h), a^{[0,h]}= 1$ is known as a factorial power of *a* with increment *h*. For $\alpha=0$, the operator (1.3) reduces to Baskakov operators [4].

In 1989, Razi [5] studied convergence properties of Stancu-Kantorovich operators based on Pólya-Eggenberger distribution. Very recently, Deo *et al.* [6] introduced a Stancu-Kantorovich operators based on inverse Pólya-Eggenberger distribution and studied some of its convergence properties. For some other relevant research in this direction we refer the reader to [7–9].

Now, for $\alpha=\frac{1}{n}$, we get a special case of Stancu-Baskakov operators (1.3) defined as

1.4 $$ V_{n}^{\langle \frac{1}{n}\rangle }(f;x)=\frac{(2n-1)!}{(n-1)!}\sum _{k=0}^{\infty }{\binom{n+k-1}{k}} \frac{(nx)_{k}}{(n+nx)_{n+k}}f \biggl(\frac{k}{n} \biggr), $$

where $(a)_{n}:= a^{[n,-1]}=a(a+1)\cdots(a+(n-1))$ is called the Pochhammer symbol.

For the Lupas operator, given by

1.5 $$\begin{aligned} L_{n}(f;x)=\sum_{k=0}^{\infty} {\binom{{n+k-1}}{k}}\frac {t^{k}}{(1+t)^{n+k}}f \biggl(\frac{k}{n} \biggr), \end{aligned}$$

let $\mu_{n,m}(x)= L_{n}(t^{m};x), m\in\mathbb{N}\cup\{0\}$ be the *m*th order moment.

### Lemma 1

*For the function* $\mu_{n,m}(x)$, *we have* $\mu_{n,0}(x)=1$ *and we have the recurrence relation*

1.6 $$\begin{aligned} x(1+x)\mu_{n,m}^{\prime}(x)= n\mu_{n,m+1}(x)-nx \mu_{n,m}(x),\quad m\in \mathbb{N}\cup\{0\}, \end{aligned}$$

*where* $\mu_{n,m}^{\prime}(x)$ *is the derivative of* $\mu_{n,m}(x)$.

### Proof

On differentiating $\mu_{n,m}(x)$ with respect to *x*, the proof of the recurrence relation easily follows; hence the details are omitted. □

### Remark 1

From Lemma 1, we have

$$ \mu_{n,1}(x)=x,\qquad \mu_{n,2}(x)=\frac{x+(n+1)x^{2}}{n}, \qquad \mu_{n,3}(x)=\frac{(n+1)(n+2)x^{3}+3(n+1)x^{2}+x}{n^{2}}. $$

The values of the Stancu-Baskakov operators (1.4) for the test functions $e_{i}(t)=t^{i}$, $i=0,1,2$, are given in the following lemma.

### Lemma 2

[10]

*The Stancu*-*Baskakov operators* (1.4) *verify*:

(i) $V_{n}^{\langle \frac{1}{n}\rangle }(1;x)= 1$,
(ii) $V_{n}^{\langle \frac{1}{n}\rangle }(t;x)= \frac{n x}{n-1}$,
(iii) $V_{n}^{\langle \frac{1}{n}\rangle }(t^{2};x)= \frac{n^{2}}{(n-1)(n-2)} [x^{2}+\frac{x(x+1)}{n}+\frac{1}{n}(1-\frac{1}{n})x ]$.
(iv) $V_{n}^{\langle \frac{1}{n}\rangle }(t^{3};x)= \frac{n^{3}}{(n-1)(n-2)(n-3)} [\frac{(n+1)(n+2)}{n^{2}}x^{3}+\frac{3(2n^{2}+n-1)}{n^{3}}x^{2}+\frac {(2n-1)(3n-1)}{n^{4}}x ]$
(v) $V_{n}^{\langle \frac{1}{n}\rangle }(t^{4};x)= \frac{n^{4}}{(n-1)(n-2)(n-3)(n-4)} [\frac{(n+1)(n+2)(n+3)}{n^{3}}x^{4}+\frac{6(n+1)(n+2)(2n-1)}{n^{4}}x^{3}+\frac{6(6n^{3}+n^{2}-4n+1)}{n^{5}}x^{2}+ \frac {26n^{2}-27n+7}{n^{5}}x ]$.

### Proof

The identities (i)-(iii) are proved in [10], hence we give the proof of the identity (iv). The identity (v) can be proved in a similar manner.

We have

$$\begin{aligned} V_{n}^{\langle {\frac{1}{n}}\rangle }\bigl(t^{3};x\bigr) =& \frac{(2n-1!)}{(n-1)!}\sum_{k=0}^{\infty} {\binom{(n+k-1)}{{k}}}\frac{(n x)_{k}}{(n+nx)_{n+k}} \biggl(\frac {k}{n} \biggr)^{3} \\ =& \frac{1}{B(n x,n)} \int_{0}^{\infty}\frac{t^{nx-1}}{(1+t)^{nx+n}} \mu_{n,3}(t)\,dt, \end{aligned}$$

where $B(nx,n)$ is the Beta function.

Therefore using Remark 1, we get

$$\begin{aligned} V_{n}^{\langle {\frac{1}{n}}\rangle }\bigl(t^{3};x\bigr) =& \frac{1}{B(n x,n)} \int_{0}^{\infty }\frac{t^{nx-1}}{(1+t)^{nx+n}} \biggl[ \frac{(n+1)(n+2)t^{3}+3(n+1)t^{2}+t}{n^{2}} \biggr]\,dt. \end{aligned}$$

Now, by a simple calculation, we get the required result. □

As a consequence of Lemma 2, we obtain the following.

### Lemma 3

*For the Stancu*-*Baskakov operator* (1.4), *the following equalities hold*:

(i) $V_{n}^{\langle \frac{1}{n}\rangle }((t-x);x)= \frac{x}{n-1}$,
(ii) $V_{n}^{\langle \frac{1}{n}\rangle }((t-x)^{2};x)= \frac{2nx(x+1)+(2x-1)x}{(n-1)(n-2)} $,
(iii) $V_{n}^{\langle \frac{1}{n}\rangle }((t-x)^{4};x)= \frac{1}{n(n-1)(n-2)(n-3)(n-4)} [ 12n(n^{2}-13n+2)x^{3}(x+1)+12n(n^{2}+8n-13)x^{2}(x+1)+(26n^{2}+48n-22)x(x+1)+(29-75n)x ]$.

Let $0\leq r_{n}(x)\leq1$ be a sequence of continuous functions for each $x\in[0,1]$ and $n\in\mathbb{N}$. Using this sequence $r_{n}(x)$, for any $f\in C[0,1]$, King [11] proposed the following modification of the Bernstein polynomial for a better approximation:

$$ \bigl((B_{n}f)\circ r_{n}\bigr) (x)= \sum _{k=0}^{n}f \biggl(\frac{k}{n} \biggr) { \binom{n}{k}} \bigl(r_{n}(x)\bigr)^{k} \bigl(1-r_{n}(x)\bigr)^{n-k}. $$

Gonska *et al.* [12] introduced a sequence of King-type operators $D_{n}^{\tau}: C[0,1]\rightarrow C[0,1]$ defined as

$$ D_{n}^{\tau}f= (B_{n}f) \circ (B_{n} \tau)^{-1} \circ \tau, $$

where $\tau\in C[0,1]$ such that $\tau(0)=0, \tau(1)=1$ and $\tau^{\prime}(x)>0$ for each $x\in[0,1]$ and studied global smoothness preservation, the approximation of decreasing and convex functions, the validity of a Voronovskaja-type theorem and a recursion formula generalizing a corresponding result for the classical Bernstein operators.

Motivated by the above work, in the present paper we introduce modified Stancu-Baskakov operators based on a function $\tau(x)$ and obtain the rate of approximation of these operators with the help of Peetre’s K-functional and the Ditzian-Totik modulus of smoothness. Also, we prove a quantitative Voronovskaja-type theorem by using the first order Ditzian-Totik modulus of smoothness.

Throughout this paper, we assume that *C* denotes a constant not necessarily the same at each occurence.

## Modified Stancu-Baskakov operators

Let $\tau(x)$ be continuously differentiable ∞ times on $[0, \infty)$, such that $\tau(0)=0, \tau^{\prime}(x)>0$ and $\tau^{\prime \prime}(x)$ is bounded for all $x\in[0,\infty)$. We introduce a sequence of Stancu-Baskakov operators for $f\in C_{B}[0, \infty)$, the space of all continuous and bounded functions on $[0,\infty)$, endowed with the norm $\| f \|= \sup_{x\in[0,\infty)}| f(x)|$, by

2.1 $$ V_{n}^{\langle \frac{1}{n}, \tau\rangle }(f;x)= \sum _{k=0}^{\infty} p_{n,k}^{\langle \frac{1}{n}, \tau\rangle }(x) \bigl(f \circ\tau^{-1}\bigr) \biggl(\frac{k}{n} \biggr),\quad x\in[0, \infty), $$

where

$$ p_{n,k}^{\langle \frac{1}{n}, \tau\rangle }(x)=\frac{(2n-1)!}{(n-1)!}{\binom {n+k-1}{k}}\frac{ (n\tau(x) )_{k}}{ (n(1+\tau(x)) )_{n+k}}. $$

### Lemma 4

*The operator defined by* (2.1) *satisfies the following equalities*:

(i) $V_{n}^{\langle \frac{1}{n},\tau\rangle }(1;x)= 1$,
(ii) $V_{n}^{\langle \frac{1}{n},\tau\rangle }(\tau(t);x)= \frac{n \tau(x)}{n-1}$,
(iii) $V_{n}^{\langle \frac{1}{n},\tau\rangle }(\tau^{2}(t);x)= \frac{n^{2}}{(n-1)(n-2)} [\tau^{2}(x)+\frac{\tau(x)(\tau(x)+1)}{n}+\frac{1}{n}(1- \frac{1}{n})\tau(x) ]$,
(iv) $V_{n}^{\langle \frac{1}{n}\rangle }(\tau^{3}(t);x)= \frac{n^{3}}{(n-1)(n-2)(n-3)} [\frac{(n+1)(n+2)}{n^{2}}\tau^{3}(x)+\frac {3(2n^{2}+n-1)}{n^{3}}\tau^{2}(x)+\frac{(2n-1)(3n-1)}{n^{4}}\tau(x) ]$,
(v) $V_{n}^{\langle \frac{1}{n}\rangle }(\tau^{4}(t);x)= \frac{n^{4}}{(n-1)(n-2)(n-3)(n-4)} [\frac{(n+1)(n+2)(n+3)}{n^{3}}\tau^{4}(x)+\frac{6(n+1)(n+2)(2n-1)}{n^{4}}\tau^{3}(x) +\frac{6(6n^{3}+n^{2}-4n+1)}{n^{5}}\tau^{2}(x)+ \frac{26n^{2}-27n+7}{n^{5}}\tau(x) ]$.

### Proof

The proof of lemma is straightforward on using Lemma 2. Hence we omit the details. □

Let the *m*th order central moment for the operators given by (2.1) be defined as

$$ \mu_{n,m}^{\tau}(x)= V_{n}^{\langle \frac{1}{n},\tau\rangle }\bigl( \bigl(\tau(t)-\tau(x)\bigr)^{m};x\bigr). $$

### Lemma 5

*For the central moment operator* $\mu_{n,m}^{\tau}(x)$, *the following equalities hold*:

(i) $\mu_{n,1}^{\tau}(x)= \frac{\tau(x)}{n-1}$,
(ii) $\mu_{n,2}^{\tau}(x)= \frac{2n\phi^{2}_{\tau}(x)+(2\tau(x)-1)\tau(x)}{(n-1)(n-2)} $,
(iii) $\mu_{n,4}^{\tau}(x)= \frac{1}{n(n-1)(n-2)(n-3)(n-4)} [ 12n(n^{2}-13n+2)\tau^{2}(x)\phi^{2}_{\tau}(x)+12n(n^{2}+8n-13)\tau(x)\phi ^{2}_{\tau}(x)+(26n^{2}+48n-22)\phi^{2}_{\tau}(x)+(29-75n)\tau(x) ]$,

*where* $\phi_{\tau(x)}^{2}(x)=\tau(x)(\tau(x)+1)$.

### Proof

Using the definition (2.1) of the modified Stancu-Baskakov operators and Lemma 4, the proof of the lemma easily follows. Hence, the details are omitted. □

Let

$$ W^{2}= \bigl\{ g\in C_{B}[0,\infty): g^{\prime}, g^{\prime\prime}\in C_{B}[0,\infty) \bigr\} . $$

For $f\in C_{B}[0,\infty)$ and $\delta>0$, the Peetre *K*-functional [13] is defined by

$$ K(f;\delta)= \inf_{g\in W^{2}} \bigl\{ \|f-g\|+\delta\| g\|_{W^{2}}\bigr\} , $$

where

$$ \| g\|_{W^{2}}=\|g\|+\bigl\| g^{\prime}\bigr\| +\bigl\| g^{\prime\prime}\bigr\| . $$

From [14], Proposition 3.4.1, there exists a constant $C > 0$ independent of *f* and *δ* such that

2.2 $$\begin{aligned} K(f;\delta)\leq C \bigl(\omega_{2}(f;\sqrt{\delta})+ \min\{1,\delta\}\|f\| \bigr), \end{aligned}$$

where $\omega_{2}$ is the second order modulus of smoothness of $f\in C_{B}[0,\infty)$ and is defined as

$$ \omega_{2}(f;{\delta})=\sup_{0< | h|\leq{\delta}} \sup _{x,x+2h\in [0,\infty)}\bigl|f(x+2h)-2f(x+h)+f(x)\bigr|. $$

In the following, we assume that $\inf_{x\in[0,\infty)}\tau^{\prime}(x)\geq a, a\in\mathbb{R}^{+}:=(0,\infty)$.

Next, we recall the definitions of the Ditzian-Totik first order modulus of smoothness and the *K*-functional [15]. Let $\phi_{\tau}(x):= \sqrt {\tau(x)(1 + \tau(x))}$ and $f\in C_{B}[0,\infty)$. The first order modulus of smoothness is given by

$$ \omega_{\phi_{\tau} }(f;t) = \sup_{0< h\leq t} \biggl\{ \biggl\vert f \biggl(x + \frac{h\phi_{\tau}(x)}{2} \biggr) - f \biggl(x - \frac{h\phi_{\tau}(x)}{2} \biggr)\biggr\vert ,x\pm\frac{h\phi_{\tau}(x)}{2} \in [ 0, \infty) \biggr\} . $$

Further, the appropriate *K*-functional is defined by

$$\begin{aligned} {K}_{\phi_{\tau} }(f;t)=\inf_{g\in W_{\phi_{\tau}}[0,\infty)}\bigl\{ \|f-g\|+t\bigl\| \phi_{\tau} g^{\prime}\bigr\| \bigr\} \quad (t>0), \end{aligned}$$

where $W_{\phi_{\tau} }[0,\infty)=\{g:g\in AC_{\mathrm{loc}}[0,\infty),\|\phi _{\tau} g^{\prime}\|<\infty\}$ and $g\in AC_{\mathrm{loc}}[0,\infty)$ means that *g* is absolutely continuous on every interval $[a,b]\subset[0,\infty)$. It is well known [15], p.11, that there exists a constant $C>0$ such that

2.3 $$\begin{aligned} {K}_{\phi_{\tau}}(f;t)\leq C\omega_{\phi_{\tau} }(f;t). \end{aligned}$$

### Theorem 1

*If* $f\in C_{B}[0,\infty)$, *then*

$$ \bigl\| V_{n}^{\langle \frac{1}{n},\tau\rangle }f\bigr\| \leq\| f\|. $$

### Proof

By the definition of the modified Stancu-Baskakov operators (2.1) and using Lemma 4 we have

$$ \bigl\vert V_{n}^{\langle \frac{1}{n},\tau\rangle }(f;x) \bigr\vert \leq \sum_{k=0}^{n}p_{n,k}^{\langle \frac{1}{n},\tau\rangle }(x) \biggl\vert \bigl(f\circ \tau^{-1} \bigr) \biggl(\frac{k}{n} \biggr)\biggr\vert \leq\bigl\| f\circ \tau^{-1} \bigr\| V_{n}^{\langle \frac{1}{n},\tau\rangle }(1;x)= \|f\|, $$

for every $x\in[0,\infty)$. Hence the required result is immediate. □

### Theorem 2

*Let* $f\in C_{B}[0,\infty)$. *Then*, *for* $n\geq3$, *there exists a constant* $C>0$ *such that*

$$ \bigl\vert V_{n}^{\langle \frac{1}{n},\tau\rangle }(f;x)- f(x) \bigr\vert \leq C \biggl\{ \omega_{2} \biggl(f;\frac{ \phi_{\tau}(x)}{\sqrt{n-2}} \biggr)+ \frac{\phi_{\tau }^{2}(x)}{n-2}\| f \| \biggr\} +\omega \biggl(f \circ \tau^{-1}; \biggl( \frac{\tau(x)}{n-1} \biggr) \biggr), $$

*on each compact subset of* $[0,\infty)$.

### Proof

Let *U* be a compact subset of $[0,\infty)$. For each $x\in U$, first we define an auxiliary operator as

2.4 $$ V_{n}^{*\langle \frac{1}{n},\tau\rangle } (f;x)= V_{n}^{\langle \frac{1}{n},\tau\rangle }(f;x)-f\circ \tau ^{-1} \biggl(\frac{n\tau(x)}{n-1} \biggr)+f(x). $$

Now, using Lemma 4, we have

$$ V_{n}^{*\langle \frac{1}{n},\tau\rangle }(1;x)=1 \quad\mbox{and}\quad V_{n}^{*\langle \frac{1}{n},\tau\rangle } \bigl(\tau(t);x\bigr)=\tau(x) \quad\mbox{hence } V_{n}^{*\langle \frac{1}{n},\tau\rangle } \bigl(\tau(t)-\tau(x);x\bigr)=0. $$

Let $g\in W^{2}$, $x\in U$ and $t\in[0, \infty)$. Then by Taylor’s expansion, and using results in [16], p.32, we get

2.5 $$\begin{aligned} g(t) =& \bigl(g\circ \tau^{-1}\bigr) \bigl(\tau(t)\bigr) \\ =& \bigl(g\circ \tau^{-1}\bigr) \bigl(\tau(x)\bigr)+ \bigl(g\circ \tau^{-1}\bigr)^{\prime}\bigl(\tau(x)\bigr) \bigl(\tau(t)-\tau(x) \bigr)+ \int_{\tau(x)}^{\tau (t)}\bigl(\tau(t)-u\bigr) \bigl(g\circ \tau^{-1}\bigr)^{\prime\prime}(u)\,du \\ =& g(x)+ \bigl(g\circ \tau^{-1}\bigr)^{\prime}\bigl(\tau(x)\bigr) \bigl(\tau(t)-\tau(x)\bigr)+ \int_{\tau(x)}^{\tau(t)}\bigl(\tau(t)-u\bigr) \frac{g^{\prime\prime}(\tau ^{-1}(u))}{[\tau^{\prime}(\tau^{-1}(u))]^{2}}\,du \\ &{}- \int_{\tau(x)}^{\tau(t)}\bigl(\tau(t)-u\bigr) \frac{g^{\prime}(\tau ^{-1}(u))\tau^{\prime\prime}(\tau^{-1}(u))}{[\tau^{\prime}(\tau^{-1}(u))]^{3}}\,du. \end{aligned}$$

Now, applying the operator $V_{n}^{*\langle \frac{1}{n},\tau\rangle }(\cdot;x)$ to both sides of the above equality, we get

2.6 $$\begin{aligned} &V_{n}^{*\langle \frac{1}{n},\tau\rangle }(g;x)-g(x) \\ &\quad= \bigl(g\circ \tau^{-1} \bigr)^{\prime}\bigl(\tau(x)\bigr)V_{n}^{*\langle \frac{1}{n},\tau\rangle }\bigl(\bigl( \tau(t)-\tau(x)\bigr);x\bigr) \\ &\qquad{}+ V_{n}^{*\langle \frac{1}{n},\tau\rangle } \biggl( \int_{\tau(x)}^{\tau(t)}\bigl( \tau(t)-u\bigr) \frac{g^{\prime\prime}(\tau^{-1}(u))}{[\tau^{\prime}(\tau^{-1}(u))]^{2}}\,du;x \biggr) \\ &\qquad{}-V_{n}^{*\langle \frac{1}{n},\tau\rangle } \biggl( \int_{\tau(x)}^{\tau(t)}\bigl(\tau (t)-u\bigr) \frac{g^{\prime}(\tau^{-1}(u))\tau^{\prime\prime}(\tau^{-1}(u))}{[\tau^{\prime}(\tau^{-1}(u))]^{3}}\,du;x \biggr) \\ &\quad= V_{n}^{\langle \frac{1}{n},\tau\rangle } \biggl( \int_{\tau(x)}^{\tau(t)}\bigl(\tau(t)-u\bigr) \frac{g^{\prime\prime}(\tau^{-1}(u))}{[\tau^{\prime}(\tau ^{-1}(u))]^{2}}\,du;x \biggr) \\ &\qquad{}- \int_{\tau(x)}^{\frac{n\tau(x)}{n-1}}\biggl(\frac{n\tau (x)}{n-1}-u\biggr) \frac{g^{\prime\prime}(\tau^{-1}(u))}{[\tau^{\prime}(\tau^{-1}(u))]^{2}}\,du \\ &\qquad{}- V_{n}^{\langle \frac{1}{n},\tau\rangle } \biggl( \int_{\tau(x)}^{\tau(t)} \bigl(\tau (t)-u \bigr) \frac{g^{\prime}(\tau^{-1}(u))\tau^{\prime\prime}(\tau^{-1}(u))}{ [\tau^{\prime}(\tau^{-1}(u))]^{3}}\,du;x \biggr) \\ &\qquad{}+ \int_{\tau(x)}^{\frac{n\tau(x)}{n-1}} \biggl(\frac{n\tau (x)}{n-1}-u \biggr)\frac{g^{\prime}(\tau^{-1}(u))\tau^{\prime\prime} (\tau^{-1}(u))}{[\tau^{\prime}(\tau^{-1}(u))]^{3}}\,du. \end{aligned}$$

Again, for each $x\in U$, we have

2.7 $$\begin{aligned} &\bigl\vert V_{n}^{*\langle \frac{1}{n},\tau\rangle }(g;x)-g(x) \bigr\vert \\ &\quad\leq\frac{1}{2}\frac{ \| g^{\prime\prime}\|}{a^{2}}V_{n}^{\langle \frac{1}{n},\tau \rangle } \bigl(\bigl(\tau(t)-\tau(x)\bigr)^{2};x \bigr)+ \frac{1}{2}\frac{\| g^{\prime\prime}\|}{a^{2}} \biggl(\frac{n\tau(x)}{n-1}-\tau (x) \biggr)^{2} \\ &\qquad{}+ \frac{1}{2}\frac{\| g^{\prime}\|\| \tau^{\prime\prime}\|}{a^{3}}V_{n}^{\langle \frac{1}{n},\tau\rangle } \bigl(\bigl(\tau(t)-\tau(x)\bigr)^{2};x \bigr)+ \frac{1}{2}\frac{\| g^{\prime}\|\|\tau^{\prime\prime}\| }{a^{3}} \biggl(\frac{n\tau(x)}{n-1}-\tau(x) \biggr)^{2} \\ &\quad= \frac{1}{2} \biggl( \frac{\| g^{\prime\prime}\| }{a^{2}}+\frac{\| g^{\prime}\|\|\tau^{\prime\prime}\|}{a^{3}} \biggr) \biggl[ V_{n}^{\langle \frac{1}{n},\tau\rangle } \bigl(\bigl(\tau(t)-\tau (x) \bigr)^{2};x \bigr)+ \biggl(\frac{\tau(x)}{n-1} \biggr)^{2} \biggr]. \end{aligned}$$

Now, using the definition of the auxiliary operators, Theorem 1 and inequality (2.7), for each $x\in U$ we have

2.8 $$\begin{aligned} &\bigl\vert V_{n}^{\langle \frac{1}{n},\tau\rangle }(f;x)-f(x) \bigr\vert \\ &\quad\leq \bigl\vert V_{n}^{*\langle \frac{1}{n},\tau\rangle }(f-g;x) \bigr\vert + \bigl\vert V_{n}^{*\langle \frac{1}{n},\tau\rangle }(g;x)-g(x) \bigr\vert \\ &\qquad{}+ \bigl| g(x)-f(x)\bigr|+\biggl| f\circ \tau^{-1} \biggl( \frac{n\tau(x)}{n-1} \biggr)- f\circ\tau^{-1}\bigl(\tau(x)\bigr)\biggr| \\ &\quad\leq 4\| f-g \|+ \frac{1}{2} \biggl( \frac{\| g^{\prime\prime}\|}{a^{2}}+ \frac{\| g^{\prime}\|\|\tau^{\prime\prime}\|}{a^{3}} \biggr) \biggl[ V_{n}^{\langle \frac{1}{n},\tau\rangle } \bigl(\bigl(\tau(t)-\tau(x)\bigr)^{2};x \bigr)+ \biggl(\frac{\tau(x)}{n-1} \biggr)^{2} \biggr] \\ &\qquad{}+ \omega \biggl(f\circ \tau^{-1}; \biggl( \frac{\tau(x)}{n-1} \biggr) \biggr). \end{aligned}$$

Let $C=\max (4,\frac{4}{a^{2}}, \frac{4}{a^{3}}\|\tau^{\prime \prime} \| )$, we get

2.9 $$\begin{aligned} \bigl\vert V_{n}^{\langle \frac{1}{n},\tau\rangle }(f;x)-f(x) \bigr\vert \leq& C \biggl(\|f-g \|+ \| g\|_{W^{2}}\frac{\phi_{\tau }^{2}(x)}{n-2} \biggr)+ \omega \biggl(f\circ \tau^{-1}; \biggl(\frac{\tau(x)}{n-1} \biggr) \biggr). \end{aligned}$$

Taking the infimum on the right side of the above inequality over all $g\in W^{2}$ and for all $x\in U$, we have

2.10 $$\begin{aligned} \bigl\vert V_{n}^{\langle \frac{1}{n},\tau\rangle }(f;x)-f(x) \bigr\vert \leq& C K \biggl( f;\frac{\phi_{\tau}^{2}(x)}{n-2} \biggr)+ \omega \biggl(f\circ \tau^{-1}; \biggl( \frac{\tau(x)}{n-1} \biggr) \biggr), \end{aligned}$$

using equation (2.2), we get the required result. □

### Theorem 3

*Let* $f\in C_{B}[0,\infty)$. *Then for every* $x\in[0,\infty )$, *and* $n\geq3$ *we have*

$$ \bigl\vert V_{n}^{\langle \frac{1}{n},\tau\rangle }(f;x)-f(x)\bigr\vert \leq C \omega_{\phi_{\tau }} \biggl(f;\frac{\sqrt{6}c(x)}{a\sqrt{(n-2)}} \biggr). $$

### Proof

For any $g\in W_{\phi_{\tau}}[0,\infty)$, by Taylor’s expansion, we have

$$ g(t)= \bigl(g\circ \tau^{-1}\bigr) \bigl(\tau(t)\bigr)= \bigl(g\circ \tau^{-1}\bigr) \bigl(\tau(x)\bigr)+ \int_{\tau (x)}^{\tau (t)}\bigl(g\circ \tau^{-1} \bigr)^{\prime}(u)\,du. $$

Applying the operator $V_{n}^{\langle \frac{1}{n},\tau\rangle }(\cdot;x)$ on both sides of the above equality, we get

2.11 $$ \bigl\vert V_{n}^{\langle \frac{1}{n},\tau\rangle }(g;x)-g(x) \bigr\vert = \biggl| V_{n}^{\langle \frac{1}{n},\tau\rangle } \biggl( \int_{\tau(x)}^{\tau(t)}\bigl(g\circ \tau^{-1} \bigr)^{\prime}(u)\,du \biggr) \biggr|. $$

From [16], we have

2.12 $$ \begin{aligned}[b] \biggl| \int_{\tau(x)}^{\tau{(t)}}\bigl(g\circ \tau^{-1} \bigr)^{\prime}(u)\,du \biggr|&= \biggl| \int_{x}^{t}\frac{g^{\prime}(y)}{\tau^{\prime}(y)}\tau^{\prime}(y)\,dy \biggr|= \biggl| \int_{x}^{t} \frac{\phi_{\tau }(y)}{\phi_{\tau}(y)}\frac{g^{\prime}(y)}{\tau^{\prime}(y)} \tau^{\prime}(y)\,dy \biggr| \\ &\leq\frac{\|\phi_{\tau}g^{\prime} \|}{a} \biggl|\int_{x}^{t}\frac{\tau^{\prime}(y)}{\phi_{\tau}(y)}\,dy \biggr| \end{aligned} $$

and

2.13 $$\begin{aligned} \biggl| \int_{x}^{t}\frac{\tau^{\prime}(y)}{\phi_{\tau}(y)}\,dy \biggr| \leq& \biggl| \int_{x}^{t} \biggl( \frac{1}{\sqrt{\tau(y)}}+ \frac{1}{ \sqrt{1+\tau(y)}} \biggr)\tau^{\prime}(y)\,dy \biggr| \\ \leq& \biggl| \int_{x}^{t} \frac{1}{\sqrt{\tau(y)}} \tau^{\prime}(y)\,dy \biggr|+ \biggl| \int_{x}^{t} \frac{1}{\sqrt{1+\tau(y)}} \tau^{\prime}(y)\,dy \biggr| \\ =& 2 \bigl\{ \bigl|\sqrt{\tau(t)}-\sqrt{\tau(x)}\bigr|+ \bigl|\sqrt {1+\tau(t)}- \sqrt{1+\tau(x)} \bigr| \bigr\} \\ < & 2\bigl|\tau(t)-\tau(x)\bigr| \biggl( \frac{1}{\sqrt{\tau(x)}}+\frac {1}{\sqrt{1+\tau(x)}} \biggr) \\ =& \frac{ 2|\tau(t)-\tau(x)|}{\sqrt{\tau(x)(1+\tau(x))}} \bigl( \sqrt{1+\tau(x)}+\sqrt{\tau(x)} \bigr) \\ =& \frac{ 2|\tau(t)-\tau(x)|}{\sqrt{\tau(x)(1+\tau(x))}} c(x) \\ =& \frac{ 2c(x)|\tau(t)-\tau(x)|}{\phi_{\tau}(x)}. \end{aligned}$$

Now, from equations (2.12)-(2.13) and using the Cauchy-Schwarz inequality, we obtain

2.14 $$\begin{aligned} \bigl\vert V_{n}^{\langle \frac{1}{n},\tau\rangle }(g;x)-g(x) \bigr\vert \leq& \frac{2c(x) \|\phi_{\tau}g^{\prime} \|}{a \phi_{\tau}(x)}V_{n}^{\langle \frac {1}{n},\tau\rangle } \bigl( \bigl\vert \tau(t)-\tau(x)\bigr\vert ;x \bigr) \\ \leq& \frac{2c(x)\|\phi_{\tau}g^{\prime} \|}{a \phi_{\tau}(x)}V_{n}^{\langle \frac{1}{n},\tau\rangle } \bigl(\bigl(\tau(t)- \tau (x)\bigr)^{2};x \bigr)^{\frac{1}{2}} \\ =& \frac{2c(x)\|\phi_{\tau}g^{\prime} \|}{a \phi_{\tau}(x)} \biggl[\frac{2n\phi_{\tau}^{2}(x)+(2\tau(x)-1)\tau(x)}{(n-1)(n-2)} \biggr]^{\frac{1}{2}}. \end{aligned}$$

Thus, for $f\in C_{B}[0,\infty)$ and any $g\in W_{\phi_{\tau}}[0,\infty)$, we have

2.15 $$\begin{aligned} \bigl\vert V_{n}^{\langle \frac{1}{n}, \tau\rangle }(f;x)-f(x)\bigr\vert \leq& \bigl\vert V_{n}^{\langle \frac{1}{n}, \tau\rangle }(f-g;x)\bigr\vert + \bigl\vert f(x)-g(x)\bigr\vert + \bigl\vert V_{n}^{\langle \frac{1}{n}, \tau\rangle }(g;x)-g(x) \bigr\vert \\ \leq& 2\| f-g\|+ \frac{2c(x)\| \phi_{\tau}g^{\prime} \|}{a \phi_{\tau}(x)} \biggl[\frac{2n\phi_{\tau}^{2}(x)+(2\tau(x)-1)\tau(x)}{(n-1)(n-2)} \biggr]^{\frac{1}{2}} \\ =&\frac{2c(x)\|\phi_{\tau}g^{\prime} \|}{a} \biggl[\frac {2(n+1)}{(n-1)(n-2)} \biggr]^{\frac{1}{2}}+2\| f-g \| \\ \leq& 2 \biggl\{ \| f-g\|+\frac{\sqrt{6}c(x)}{a\sqrt {(n-2)}}\bigl\| \phi_{\tau}g^{\prime} \bigr\| \biggr\} . \end{aligned}$$

Taking the infimum on the right side of the above inequality over all $g\in W_{\phi_{\tau}}[0, \infty)$, we get

$$ \bigl\vert V_{n}^{\langle \frac{1}{n},\tau\rangle }(f;x)-f(x)\bigr\vert \leq2 K_{\phi_{\tau }} \biggl(f; \frac{\sqrt{6}c(x)}{a\sqrt{(n-2)}} \biggr). $$

Finally, using equation (2.3), the theorem is immediate. □

### Theorem 4

*For any* $f\in C^{2}[0,\infty)$ *and* $x\in[0,\infty)$, *the following inequality hold*:

$$\begin{aligned} &\biggl\vert V_{n}^{\langle \frac{1}{n},\tau\rangle }(f;x)-f(x)- \frac{f^{\prime}(x)}{\tau^{\prime}(x)} \mu_{n,1}^{\tau}(x)- \frac{1}{2} \biggl[ \frac{f^{\prime\prime}(x)}{(\tau^{\prime}(x))^{2}} -f^{\prime}(x)\frac{\tau^{\prime\prime}(x)}{(\tau^{\prime}(x))^{3}} \biggr] \mu_{n,2}^{\tau}(x)\biggr\vert \\ &\quad\leq \bigl(\mu_{n,2}^{\tau}(x) \bigr)^{\frac{1}{2}} \biggl[\bigl\| \bigl(f\circ\tau^{-1} \bigr)^{\prime\prime}-g\bigr\| \bigl(\mu_{n,2}^{\tau }(x)\bigr)^{\frac{1}{2}}+ \frac{2\|\phi_{\tau}g^{\prime}\|}{a}\phi_{\tau}^{-1}(x) \bigl( \mu_{n,4}^{\tau}(x) \bigr)^{1/2} \biggr]. \end{aligned}$$

### Proof

Let $f\in C^{2}[0,\infty)$ and $x,t\in{}[0,\infty)$. Then by Taylor’s expansion, we have

$$\begin{aligned} f(t)& = \bigl( f\circ\tau^{-1} \bigr) \bigl(\tau(t)\bigr) \\ & = \bigl( f\circ\tau^{-1} \bigr) \bigl(\tau(x)\bigr)+ \bigl( f\circ \tau ^{-1} \bigr) ^{\prime}\bigl(\tau(x)\bigr) \bigl( \tau(t)- \tau(x) \bigr) +\int_{\tau(x)}^{\tau(t)}\bigl(\tau(t)-u\bigr) \bigl( f\circ\tau ^{-1} \bigr) ^{\prime\prime}(u)\,du. \end{aligned}$$

Hence,

$$\begin{aligned} & f(t)-f(x)- \bigl( f\circ\tau^{-1} \bigr) ^{\prime}\bigl(\tau(x) \bigr) \bigl( \tau (t)-\tau(x) \bigr) - \frac{1}{2} \bigl( f\circ \tau^{-1} \bigr) ^{\prime\prime}\bigl(\tau(x)\bigr) \bigl( \tau(t)- \tau(x) \bigr) ^{2} \\ &\quad = \int_{\tau(x)}^{\tau(t)}\bigl(\tau(t)-u\bigr) \bigl( f\circ\tau ^{-1} \bigr) ^{\prime\prime}(u)\,du- \int_{\tau(x)}^{\tau (t)}\bigl(\tau(t)-u\bigr) \bigl( f\circ \tau^{-1} \bigr) ^{\prime\prime}\bigl(\tau(x)\bigr)\,du \\ &\quad = \int_{\tau(x)}^{\tau(t)}\bigl(\tau(t)-u\bigr) \bigl[ \bigl( f \circ \tau^{-1} \bigr) ^{\prime\prime}(u)- \bigl( f\circ \tau^{-1} \bigr) ^{\prime\prime}\bigl(\tau(x)\bigr) \bigr]\,du. \end{aligned}$$

Applying $V_{n}^{\langle \frac{1}{n},\tau\rangle }$ to both sides of the above relation, we get

2.16 $$\begin{aligned} & \biggl\vert V_{n}^{\langle \frac{1}{n},\tau\rangle }(f;x)-f(x)- \frac{f^{\prime}(x)}{\tau^{\prime}(x)}\mu_{n,1}^{\tau}(x)- \frac{1}{2} \biggl[ \frac{f^{\prime\prime}(x)}{(\tau^{\prime }(x))^{2}}-f^{\prime }(x)\frac{\tau^{\prime\prime}(x)}{(\tau^{\prime}(x))^{3}} \biggr] \mu _{n,2}^{\tau}(x)\biggr\vert \\ & \quad=\biggl\vert V_{n}^{\langle \frac{1}{n},\tau\rangle } \biggl( \int_{\tau (x)}^{\tau(t)}\bigl(\tau(t)-u\bigr) \bigl[ \bigl( f \circ\tau^{-1} \bigr) ^{\prime \prime}(u)- \bigl( f\circ \tau^{-1} \bigr) ^{\prime\prime}\bigl(\tau(x)\bigr) \bigr]\,du;x \biggr) \biggr\vert \\ & \quad\leq V_{n}^{\langle \frac{1}{n},\tau\rangle } \biggl( \biggl\vert \int_{\tau (x)}^{\tau(t)}\bigl\vert \tau(t)-u\bigr\vert \bigl\vert \bigl( f\circ \tau^{-1} \bigr) ^{\prime\prime}(u)- \bigl( f \circ\tau^{-1} \bigr) ^{\prime\prime}\bigl(\tau(x)\bigr)\bigr\vert \,du \biggr\vert ;x \biggr) . \end{aligned}$$

For $g\in W_{\phi_{\tau}[0,\infty)}$, we have

$$\begin{aligned} & \biggl\vert \int_{\tau(x)}^{\tau(t)}\bigl\vert \tau (t)-u\bigr\vert \bigl\vert \bigl( f\circ\tau^{-1} \bigr) ^{\prime \prime }(u)- \bigl( f \circ\tau^{-1} \bigr) ^{\prime\prime}\bigl(\tau(x)\bigr)\bigr\vert \,du \biggr\vert \\ &\quad \leq\biggl\vert \int_{\tau(x)}^{\tau(t)}\bigl\vert \tau (t)-u\bigr\vert \bigl\vert \bigl( f\circ\tau^{-1} \bigr) ^{\prime \prime }(u)- \bigl( g \circ\tau^{-1} \bigr) (u)\bigr\vert \,du\biggr\vert \\ &\qquad{} +\biggl\vert \int_{\tau(x)}^{\tau(t)}\bigl\vert \tau (t)-u\bigr\vert \bigl\vert \bigl( g\circ\tau^{-1} \bigr) (u)- \bigl( g\circ \tau^{-1} \bigr) \bigl(\tau(x)\bigr)\bigr\vert \,du\biggr\vert \\ &\qquad{} +\biggl\vert \int_{\tau(x)}^{\tau(t)}\bigl\vert \tau (t)-u\bigr\vert \bigl\vert \bigl( g\circ\tau^{-1} \bigr) \bigl(\tau (x)\bigr)- \bigl( f\circ\tau^{-1} \bigr) ^{\prime\prime}\bigl(\tau(x)\bigr)\bigr\vert \,du \biggr\vert \\ &\quad =\biggl\vert \int_{x}^{t}\bigl\vert \bigl( f\circ\tau ^{-1} \bigr) ^{\prime\prime} \bigl( \tau(y) \bigr) -g(y)\bigr\vert \bigl\vert \tau(t)-\tau(y)\bigr\vert \tau^{\prime}(y)\,dy\biggr\vert \\ &\qquad{} +\biggl\vert \int_{x}^{t}\bigl\vert g(y)-g(x)\bigr\vert \bigl\vert \tau(t)-\tau(y)\bigr\vert \tau^{\prime}(y)\,dy\biggr\vert \\ &\qquad{} + \biggl\vert \int_{x}^{t}\bigl\vert g(x)- \bigl( f\circ\tau ^{-1} \bigr) ^{\prime\prime} \bigl( \tau(x) \bigr) \bigr\vert \bigl\vert \tau(t)-\tau(y)\bigr\vert \tau^{\prime}(y)\,dy\biggr\vert \\ &\quad \leq2\bigl\| \bigl( f\circ\tau^{-1} \bigr) ^{\prime\prime}-g\bigr\| \biggl\vert \int_{x}^{t}\bigl|\tau(t)-\tau(y)\bigr|\tau^{\prime }(y)\,dy \biggr\vert \\ &\qquad{}+\biggl\vert \int_{x}^{t}\biggl\vert \int_{x}^{y}\bigl|g^{\prime}(v)\bigr|\,dv\biggr\vert \bigl| \tau(t)-\tau(y)\bigr|\tau ^{\prime }(y)\,dy\biggr\vert \\ &\quad \leq\bigl\| \bigl( f\circ\tau^{-1} \bigr) ^{\prime\prime}-g\bigr\| \bigl( \tau (t)-\tau(x)\bigr)^{2}+\bigl\| \phi_{\tau}g^{\prime}\bigr\| \biggl\vert \int_{x}^{t}\biggl\vert \int_{x}^{y}\frac{dv}{\phi_{\tau}(v)}\biggr\vert \bigl|\tau(t)-\tau(y)\bigr|\tau^{\prime}(y)\,dy\biggr\vert . \end{aligned}$$

Using the inequality

$$ \frac{|y-v|}{v(1+v)}\leq\frac{|y-x|}{x(1+x)},\quad x< v< y, $$

we can write

$$ \frac{|\tau(y)-\tau(v)|}{\tau(v)(1+\tau(v))}\leq\frac{|\tau(y)-\tau(x)|}{\tau(x)(1+\tau(x))}. $$

Therefore,

2.17 $$\begin{aligned} & \biggl\vert \int_{\tau(x)}^{\tau(t)}\bigl\vert \tau (t)-u\bigr\vert \bigl\vert \bigl( f\circ\tau^{-1} \bigr) ^{\prime \prime }(u)- \bigl( f \circ\tau^{-1} \bigr) ^{\prime\prime}\bigl(\tau(x)\bigr)\bigr\vert \,du \biggr\vert \\ &\quad\leq\bigl\| \bigl( f\circ\tau^{-1} \bigr) ^{\prime\prime }-g\bigr\| \bigl(\tau(t)-\tau(x)\bigr)^{2} \\ &\qquad{} +\bigl\| \phi_{\tau}g^{\prime}\bigr\| \biggl\vert \int_{x}^{t}\biggl\vert \int_{x}^{y} \frac{|\tau(y)-\tau (x)|^{1/2}}{\tau^{\prime}(v)\phi_{\tau}(x)}\cdot \frac{\tau ^{\prime}(v)}{|\tau(y)-\tau(v)|^{1/2}}\,dv\biggr\vert \bigl|\tau(t)-\tau(y)\bigr|\tau ^{\prime }(y)\,dy \biggr\vert \\ & \quad\leq\bigl\| \bigl( f\circ\tau^{-1} \bigr) ^{\prime\prime}-g\bigr\| \bigl( \tau (t)-\tau(x)\bigr)^{2} \\ &\qquad{}+2 \frac{\|\phi_{\tau}g^{\prime}\|}{a}\phi_{\tau}^{-1}(x) \biggl\vert \int_{x}^{t}\bigl|\tau(y)-\tau (x)\bigr| \bigl|\tau(t)-\tau(y)\bigr| \tau^{\prime}(y)\,dy\biggr\vert \\ &\quad \leq\bigl\| \bigl( f\circ\tau^{-1} \bigr) ^{\prime\prime}-g\bigr\| \bigl( \tau (t)-\tau(x)\bigr)^{2}+2 \frac{\|\phi_{\tau}g^{\prime}\|}{a}\phi_{\tau}^{-1}(x) \biggl\vert \int_{x}^{t}\bigl(\tau(t)-\tau (x) \bigr)^{2}\tau^{\prime}(y)\,dy\biggr\vert \\ & \quad\leq\bigl\| \bigl( f\circ\tau^{-1} \bigr) ^{\prime\prime}-g\bigr\| \bigl( \tau (t)-\tau(x)\bigr)^{2}+2 \frac{\|\phi_{\tau}g^{\prime}\|}{a}\phi_{\tau}^{-1}(x)\bigl|\tau(t)-\tau(x)\bigr|^{3}. \end{aligned}$$

Now combining equations (2.16)-(2.17), applying Lemma 3 and the Cauchy-Schwarz inequality, we get

$$\begin{aligned} & \biggl\vert V_{n}^{\langle \frac{1}{n},\tau\rangle }(f;x)-f(x)- \frac{f^{\prime}(x)}{\tau^{\prime}(x)} \mu_{n,1}^{\tau}(x)- \frac{1}{2} \biggl[ \frac{f^{\prime\prime}(x)}{(\tau^{\prime }(x))^{2}}-f^{\prime }(x)\frac{\tau^{\prime\prime}(x)}{(\tau^{\prime}(x))^{3}} \biggr] \mu _{n,2}^{\tau}(x)\biggr\vert \\ &\quad \leq\bigl\| \bigl(f\circ\tau^{-1}\bigr)^{\prime\prime}-g\bigr\| V_{n}^{\langle \frac {1}{n},\tau\rangle } \bigl( \bigl(\tau(t)-\tau(x)\bigr)^{2};x \bigr) +2 \frac{\| \phi_{\tau}g^{\prime}\|}{a}\phi_{\tau}^{-1}(x)V_{n}^{\langle \frac {1}{n},\tau\rangle } \bigl( \bigl|\tau(t)-\tau(x)\bigr|^{3};x \bigr) \\ &\quad \leq \bigl\| \bigl( f\circ\tau^{-1} \bigr) ^{\prime\prime }-g\bigr\| V_{n}^{\langle \frac{1}{n},\tau\rangle } \bigl( \bigl(\tau(t)-\tau (x) \bigr)^{2};x \bigr) \\ &\qquad{} + \frac{2\|\phi_{\tau}g^{\prime}\|}{a}\phi_{\tau }^{-1}(x) \bigl[ V_{n}^{\langle \frac{1}{n},\tau\rangle } \bigl( \bigl(\tau(t)-\tau (x) \bigr)^{2};x \bigr) \bigr] ^{1/2} \bigl[ V_{n}^{\langle \frac{1}{n},\tau\rangle } \bigl( \bigl(\tau(t)-\tau(x)\bigr)^{4};x \bigr) \bigr] ^{1/2} \\ &\quad = \bigl\| \bigl( f\circ\tau^{-1} \bigr) ^{\prime\prime }-g\bigr\| \mu_{n,2}^{\tau}(x)+ \frac{2\|\phi_{\tau }g^{\prime}\|}{a}\phi_{\tau}^{-1}(x) \bigl( \mu_{n,2}^{\tau }(x) \bigr) ^{1/2} \bigl( \mu_{n,4}^{\tau}(x) \bigr) ^{1/2} \\ &\quad = \bigl( \mu_{n,2}^{\tau}(x) \bigr) ^{\frac{1}{2}} \biggl[ \bigl\| \bigl( f\circ\tau^{-1} \bigr) ^{\prime\prime}-g\bigr\| \bigl(\mu _{n,2}^{\tau}(x)\bigr)^{\frac{1}{2}}+ \frac{2\|\phi_{\tau }g^{\prime}\|}{a} \phi_{\tau}^{-1}(x) \bigl( \mu_{n,4}^{\tau }(x) \bigr) ^{1/2} \biggr] . \end{aligned}$$

This completes the proof of the theorem. □
